# Serum irisin levels correlated to peritoneal dialysis adequacy in nondiabetic peritoneal dialysis patients

**DOI:** 10.1371/journal.pone.0176137

**Published:** 2017-04-26

**Authors:** Zhijun Tan, Zengchun Ye, Jun Zhang, Yanru Chen, Cailian Cheng, Cheng Wang, Xun Liu, Tanqi Lou, Hui Peng

**Affiliations:** Division of Nephrology, Department of Medicine, Third Affiliated Hospital of Sun Yat-sen University, Guangzhou, Guangdong, China; University of Utah School of Medicine, UNITED STATES

## Abstract

**Background:**

Irisin is a recently discovered myokine thought to be involved in multiple metabolism abnormalities in most dialysis patients. However, the myokine has not been thoroughly studied in peritoneal dialysis. This study aimed to evaluate serum irisin levels and establish their relation to dialysis adequacy, insulin resistance, and bone metabolism status in patients on peritoneal dialysis.

**Methods:**

A total of 59 nondiabetic prevalent peritoneal dialysis patients and 52 age- and sex-matched healthy controls were enrolled in this cross-sectional study. Serum irisin concentration was assessed by enzyme-linked immunosorbent assay. The correlations between serum irisin and dialysis adequacy, clinical, and metabolic variables were investigated.

**Results:**

Serum irisin levels were lower in nondiabetic peritoneal dialysis patients (17.02ng/ml) compared with healthy controls (22.17ng/ml, P<0.001). Multivariate regression analysis revealed that fasting glucose levels were correlated inversely with serum irisin levels in peritoneal dialysis patients. Serum irisin levels were associated with neither insulin resistance nor bone metabolism in our patients. Serum irisin levels were positively associated with peritoneal Kt/V_urea_ (β = 4.933, 95% confidence interval [CI] = 0.536–9.331, P = 0.029) and peritoneal C_Cr_ (β = 0.259, 95% CI = 0.053–0.465, P = 0.015) among peritoneal dialysis patients.

**Conclusions:**

The study demonstrated that non-diabetic peritoneal dialysis patients have lower serum irisin levels, and the levels were correlated with peritoneal dialysis adequacy, indicating adequate dialysis may improve irisin secretion. Additional studies are needed to provide a confirmation.

## Introduction

Irisin was identified in 2012 as an exercise-induced peptide released from muscle. As the cleavage product of fibronectin type III domain containing protein 5 (FNDC5), irisin activates subcutaneous adipose tissue by increasing the expression of uncoupling protein 1, which leads to energy dissipation by thermogenesis and improvements in glucose tolerance and weight loss [1]. Thus, irisin is a promising agent for the treatment of human metabolic diseases, including diabetes and obesity.

Since energy imbalance is common among patients with end stage renal disease (ESRD) [2, 3], altered circulating irisin levels may occur in patients with ESRD and result in energy dysfunction. Indeed, studies found that circulating irisin levels were significantly lower in non-dialysis chronic kidney disease (CKD) and hemodialysis patients, compared with those of healthy individuals [4–7]. Recently, lower serum irisin levels were also found in peritoneal dialysis (PD) patients compared with healthy controls, and it was suggested that higher peritoneal Kt/V_urea_ (urea clearance expressed as Kt/V) was associated with lower irisin levels [8, 9]. However, diabetes patients were not excluded from their studies’ PD groups despite multiple studies showing that serum irisin levels are lower in diabetic than nondiabetic individuals [10, 11]. Moreover, previous studies evaluated the association between residue renal function (RRF) and serum irisin levels without adjusting for the creatinine clearance of peritoneal dialysis or knowledge of whether irisin levels were influenced by dialysis adequacy [8].

Since irisin is a myokine excreted by muscle for the regulation of energy metabolism, there may be a correlation between serum irisin and protein-energy wasting (PEW) in ESRD patients. Insulin resistance, which is prevalent in ESRD patients [12], may contribute to PEW [3]. PEW is defined as a decline in body protein mass and energy stores [13] and occurs in 18–75% of long-term dialysis patients [14]. PEW is linked to impaired physical and cognitive function, fractures due to bone loss, and increased all-cause mortality [2]. In this study, we assessed whether an association exists between irisin and PEW in PD patients.

Recently, irisin was shown to increase cortical bone mass and strength in mice by stimulating bone formation [15]. As a component of chronic kidney disease–mineral and bone disorder (CKD-MBD), renal osteodystrophy is characterized by abnormalities in bone turnover, volume, and mineralization [16, 17]. We implied that an association between irisin and CKD-MBD was observed in PD patients.

The aims of this study were to compare serum irisin levels in non-diabetic PD patients with those of healthy subjects and to investigate whether irisin is affected by dialysis adequacy. We also aimed to determine whether associations exist among serum irisin levels, PEW, insulin resistance, and CKD-MBD.

## Subjects and methods

### Subjects

This cross-sectional study screened 102 patients and enrolled 59 continuous ambulatory peritoneal dialysis (CAPD) patients between March and August 2016 from the third Affiliated Hospital of Sun Yat-sen University (Guangdong, China). A diagnosis of CKD was based on the clinical practice guidelines set by the National Kidney Foundation Disease Outcomes Quality Initiative (NKF-K/DOQI) [18]. Included in this study were patients aged ≥ 18 years old in whom CAPD was performed for more than 3 months. Patients who were pregnant and those who had diabetes, impaired liver function, heart failure, acute cardiovascular events, neurodegenerative diseases, autoimmune disease, treatment with immunosuppressive agents, glucocorticoids or catabolizing drugs, malignancy, acute infectious disease, poly cystic ovarian syndrome, hemodialysis, or bariatric surgery were excluded. Double bag systems and PD solutions (Dianeal 1.5% or 2.5% dextrose; Baxter Healthcare, Guangzhou, China) were used in all patients. CAPD involved a 2-L fluid exchange 3–5 times per day. After completing detailed clinical questionnaires and routine urine and blood tests to exclude CKD and the conditions described above, 52 age- and sex-matched healthy individuals were also included as controls. A unique identification number was provided for each participant and others had no access to information that could identify individual participants during or after data collection.

When assessing the relationship between irisin and CKD-MBD, the following additional exclusion criteria were applied: biliary obstructive disease, Cushing's syndrome, primary hyperparathyroidism, hypoparathyroidism, hyperthyroidism, rheumatologic diseases, consumption of aluminum-containing drugs, teriparatide and denosumab, paraplegia, genetic diseases including Marfan syndrome, prior musculoskeletal injuries or surgical procedures within 3 months prior to the study, or severe acidosis and acute pancreatitis. Collectively, 59 PD patients were enrolled in the study. The study protocol was approved by the ethics committee at the third Hospital of Sun Yat-Sen University. Written informed consent was provided by all participants.

### Analytic procedures and biochemical analyses

Blood samples and data collection took place between March and August 2016. Blood samples were collected in vacutainer tubes without anticoagulant after fasting for at least 8h. Following centrifugation, serum was collected and stored with aprotintin at -80°C until analysis. Medical history, demographic data, and routine biochemical parameters, including levels of triglycerides [TGs], total cholesterol [TC], HDL cholesterol [HDL-C], LDL cholesterol [LDL-C], blood urea nitrogen [BUN], serum creatinine, and albumin [ALB], were also obtained. Experimental data were obtained using the Hitachi 7180 biochemistry analyzer (Japan), and insulin levels were measured by chemiluminescence (ADVIA Centaur XP: Siemens). The estimated glomerular filtration rate was calculated based on the Chronic Kidney Disease Epidemiology Collaboration creatinine equation [19] and the modified Modification of Diet in Renal Disease formula for Chinese patients [20]. The Geriatric nutritional risk index (GNRI) was calculated using the following formula: GNRI = [1.489 × ALB (g/L)] + [41.7 × (body weight/ideal body weight)]. Ideal body weight was calculated from the Lorentz equations (WLo) [21]. Insulin resistance was estimated using the homeostatic model assessment of insulin resistance (HOMA-IR) equation: HOMA-IR = fasting serum insulin (mU/L) × fasting serum glucose (mmol/L) / 22.5 [12]. Additionally, 24-h urine and dialysate were collected to calculate weekly kidney and peritoneal Kt/V_urea_ and weekly kidney and peritoneal creatinine clearance (kidney C_Cr_ and peritoneal C_Cr_, respectively). Serum irisin levels were measured by ELISA using commercial kits (EK-067-29, Phoenix Pharmaceutical, Burlingame, CA, USA) and the intra- and inter-assay variations were <10% and <15%, respectively. The experiments were conducted on April 27, June 12 and August 27, 2016, respectively.

### Statistical analysis

SPSS Software, version 13.0 was used for all statistical analyses. The results were presented as the means ± standard deviation or medians with interquartile ranges according to the sample distributions. Categorical variables were given as frequencies (percentages). The Student’s t-test was used to compare differences between the two groups for normally distributed data, while the Mann-Whitney U test was used for non-normal data. Categorical data were compared using the Chi-square test. Correlations were expressed as Pearson’s correlation coefficients for two continuous variables, and Spearman rank correlations were used for non-normally distributed variables. Univariate linear regression analyses were performed to evaluate the determinants of serum irisin levels. Multiple stepwise linear regression models were employed to select variables independently related to serum irisin concentrations. All analyses were two-tailed, and a p<0.05 was considered statistically significant.

## Results

### Baseline characteristics

**Fig 1:**
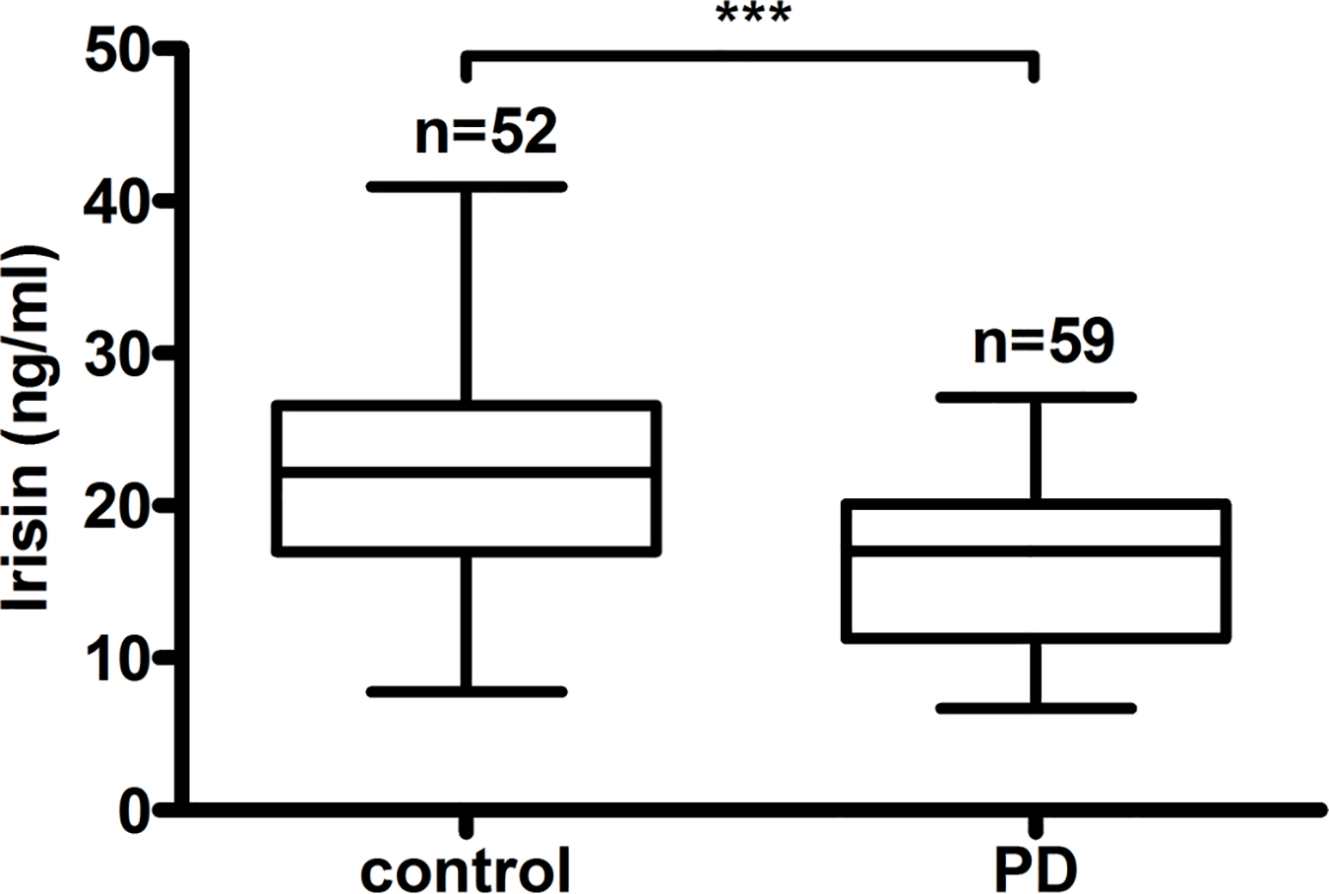
Irisin levels in PD patients and healthy controls. The box represents median and interquartile ranges, and error bars show the minimum and the maximum.***P<0.001.

The demographic and clinical characteristics of the 59 PD patients and 52 healthy controls are summarized in Table 1. The mean ages of the PD patients and healthy controls were 47.5±11.6 years and 44.3±15.0 years, respectively. Male patients and controls accounted for 57.6% and 57.7% of individuals in each group, respectively. No differences in age, sex, proportion of smokers or drinkers, or TC, TG or HDL-C levels were observed between the two groups. PD patients had a lower BMI and serum levels of fasting glucose, ALB, and LDL-C and higher blood pressure and pulse pressures, levels of BUN, serum creatinine and uric acid, utilization ratios of angiotensin-converting enzyme inhibitors/angiotensin II receptor blockers and statins compared with those in healthy controls (all p< 0.05). PD patients had a lower GNRI than that of healthy controls, and serum irisin levels were significantly lower in the nondiabetic PD group (17.02 (11.27–20.09) ng/ml) compared with the control group (22.17 (17.00–26.57) ng/ml, P<0.001) (Fig 1).

**Table 1:** Baseline demographic and clinical parameters of subjects.

	PD patients (n = 59)	Healthy subjects (n = 52)	P-value
Male (%)	34(57.6%)	30(57.7%)	0.994
Age (years)	47.5±11.6	44.3±15.0	0.215
Smoker (%)	7(11.9%)	12(23.1%)	0.118
Alcohol intake (%)	2(3.4%)	6(11.5%)	0.197
Body mass index (kg/m^2^)	20.99(19.76–22.63)	22.18(20.09–24.70)	0.043
Systolic BP (mmHg)	140(127–158)	117.5(111–125)	<0.001
Diastolic BP (mmHg)	90(80–100)	73(67–80)	<0.001
Pulse pressure (mmHg)	55(41–62)	42.5(38–56)	0.007
Fasting glucose (mmol/L)	4.4±0.5	4.8±0.6	<0.001
BUN (mmol/L)	18.0(13.9–21.1)	5.0(4.2–5.5)	<0.001
Creatinine (umol/L)	1112(837–1475)	71(63–81)	<0.001
eGFR-EPI (ml/min/1.73m^2^) ^a^	-	103.00(93.00–110.50)	**-**
eGFR-MDRD (ml/min/1.73m^2^) ^b^	-	103.90(96.00–116.18)	**-**
Albumin (g/L)	34.7(31.1–38.5)	39.9(39.0–42.5)	<0.001
Total cholesterol (mmol/L)	4.4(3.8–5.4)	4.5(4.1–5.2)	0.587
Triglyceride (mmol/L)	1.29(0.80–1.83)	1.03(0.76–1.56)	0.164
HDL-C (mmol/L)	1.11(0.90–1.37)	1.27(1.03–1.45)	0.052
LDL-C (mmol/L)	2.38(2.00–3.14)	2.78(2.37–3.22)	0.043
Uric acid (umol/L)	424.0(373.0–471.0)	343.0(294.3–402.0)	<0.001
ACE inhibitors/ARBs (%)	40(67.8%)	0(0%)	<0.001
Statins (%)	10(16.9%)	0(0%)	0.005
kidney C_Cr_ (L/(w*1.73m^2^BSA)) ^c^	7.99(0.00–29.32)	-	-
peritoneal C_Cr_ (L/(w*1.73m^2^BSA)) ^c^	43.46±6.68	-	-
Total C_Cr_ (L/(w*1.73m^2^BSA)) ^c^	54.79(46.51–69.03)	-	-
peritoneal Kt/V_urea_ (per week) ^c^	1.59(1.36–1.77)	-	-
kidney Kt/V_urea_ (per week) ^c^	0.21(0.00–0.57)	-	-
total Kt/V_urea_ (per week) ^c^	1.89(1.62–2.14)	-	-
GNRI	89.3(85.0–97.2)	100.7(96.7–103.9)	<0.001
iPTH (pg/ml)	441.32(284.79–708.19)	-	-
HOMA-IR	1.62±0.84	-	-

Values are given as mean ± standard deviation, median (interquartile range) or frequencies (percentages), as appropriate

PD = peritoneal dialysis; BP = blood pressure; BUN = blood urea nitrogen; eGFR = Estimated glomerular filtration rate; HDL-C = high-density lipoprotein-cholesterol; LDL-C = low-density lipoprotein-cholesterol; ACE inhibitors = angiotensin-converting enzyme inhibitors; ARBs = angiotensin II receptor blockers; Kt/V_urea_ = Urea clearance expressed as Kt/V; C_Cr_ = Creatinine clearance; GNRI = Geriatric nutritional risk index; iPTH = intact parathyroid hormone; HOMA-IR = homeostatic model assessment of insulin resistance; MDRD = Modification of Diet in Renal Disease

^a^ based on the CKD-EPI (Chronic Kidney Disease Epidemiology Collaboration) creatinine equation.

^b^ based on the the modified glomerular filtration rate estimating equation for Chinese patients with CKD: eGFR (ml/min/1.73 m^2^) = 175*(sCr)^-1.234^*(age)^-0.179^*(0.79 if female).

^c^ Kt/V_urea_ and C_Cr_ were measured in 54 PD patients.

### Correlations between serum irisin levels and other study parameters

Univariate correlations revealed no significant correlation between serum irisin levels and age, BMI, blood pressure, BUN, serum fasting glucose, creatinine, ALB, cholesterol, TGs, uric acid, GNRI or HOMA-IR in PD patients (Table 2).

**Table 2:** Univariate correlations with circulation irisin levels.

Covariate	PD patients (n = 59)		Healthy subjects (n = 52)	
	Correlation with irisin (r)	P-value	Correlation with irisin (r)	P-value
Age (years)	-0.161 ^a^	0.224	0.179	0.205
Body mass index (kg/m^2^)	-0.183 ^a^	0.165	0.053	0.709
Systolic BP (mmHg)	-0.065 ^a^	0.627	**0.298**	**0.032**
Diastolic BP (mmHg)	-0.109 ^a^	0.410	0.192 ^a^	0.173
Pulse pressure (mmHg)	-0.060 ^a^	0.649	0.250 ^a^	0.074
Fasting glucose (mmol/L)	-0.187 ^a^	0.157	-0.055	0.701
BUN (mmol/L)	-0.162 ^a^	0.222	0.215 ^a^	0.127
Creatinine (umol/L)	-0.122 ^a^	0.358	**0.312**	**0.024**
Albumin (g/L)	0.144 ^a^	0.275	0.184 ^a^	0.193
Total cholesterol (mmol/L)	0.204 ^a^	0.121	**0.301** ^a^	**0.030**
Triglyceride (mmol/L)	0.235 ^a^	0.074	**0.286** ^a^	**0.039**
HDL-C (mmol/L)	0.060 ^a^	0.654	-0.143	0.312
LDL-C (mmol/L)	-0.056 ^a^	0.676	0.209 ^a^	0.138
Uric acid (umol/L)	0.013 ^a^	0.922	**0.319**	**0.021**
GNRI	0.047 ^a^	0.726	0.195	0.165
HOMA-IR	-0.092^a^	0.490	-	-
peritoneal Kt/V_urea_ (per week) ^b^	**0.269** ^a^	**0.050**	-	-
kidney Kt/V_urea_ (per week) ^b^	-0.024 ^a^	0.862	-	-
total Kt/V_urea_ (per week) ^b^	0.255 ^a^	0.063	-	-
peritoneal C_Cr_ (L/(w*1.73m^2^BSA)) ^b^	**0.331**	**0.015**	-	-
kidney C_Cr_ (L/(w*1.73m^2^BSA)) ^b^	0.010^a^	0.945	-	-
Total C_Cr_ (L/(w*1.73m^2^BSA)) ^b^	0.131 ^a^	0.346	-	-
eGFR-EPI (ml/min/1.73m^2^) ^c^	-	-	-0.255 ^a^	0.068
eGFR-MDRD (ml/min/1.73m^2^) ^d^	-	-	-0.200 ^a^	0.156

Abbreviation are indicated in Table 1.

^a^ Spearman rank correlations were performed, others were expressed as Pearson’s correlation coefficients.

^b^ Kt/V_urea_ and C_Cr_ were measured in 54 PD patients.

^c^ based on the CKD-EPI (Chronic Kidney Disease Epidemiology Collaboration) creatinine equation.

^d^ based on the the modified glomerular filtration rate estimating equation for Chinese patients with CKD: eGFR (ml/min/1.73 m^2^) = 175*(sCr)^-1.234^*(age)^-0.179^*(0.79 if female).

Significant results (P<0.05) in **bold.**

### Relationship between serum irisin levels and dialysis adequacy

**Fig 2:**
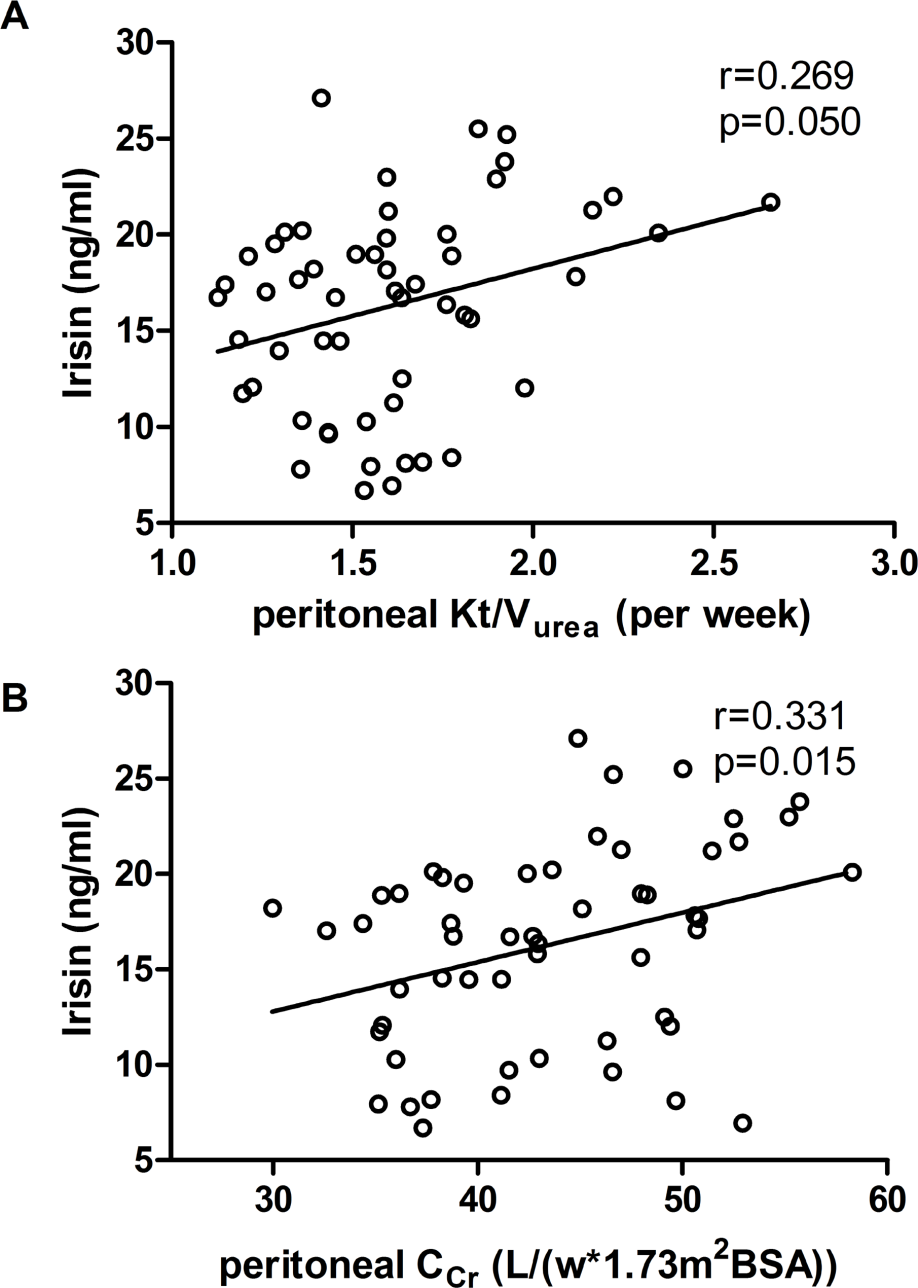
Serum irisin levels correlated with peritoneal Kt/V_urea_ and peritoneal C_Cr_. (A) Pearson’s correlation analysis was performed between serum irisin levels and peritoneal C_Cr_. (B) Spearman rank correlation was performed between serum irisin levels and peritoneal Kt/V_urea_. The continuous line indicated least-square linear regression.

Peritoneal Kt/V_urea_ (r = 0.269, p = 0.050) and peritoneal C_Cr_ (r = 0.331, p = 0.015) were positively correlated with serum irisin levels (Fig 2). However, kidney Kt/V_urea_, total Kt/V_urea_, kidney C_Cr_, and total C_Cr_ were not correlated with serum irisin levels (Table 2). Univariate linear regression analyses revealed that peritoneal Kt/V_urea_ was associated with higher serum irisin levels (β = 4.933; 95% CI, 0.536–9.331; p = 0.029). When age, sex, BMI, fasting glucose, peritoneal Kt/V_urea_, and kidney Kt/V_urea_ were included as candidate variables, multivariate stepwise linear regression analyses showed that only peritoneal Kt/V_urea_ (β = 4.933; 95% CI, 0.536–9.331; P = 0.029) was an independent factor for serum irisin levels in PD patients. Similarly, peritoneal C_Cr_ was also correlated with higher serum irisin levels in the univariate regression analysis (β = 0.259; 95% CI, 0.053–0.465; P = 0.015). Additionally, when peritoneal and kidney C_Cr_ were evaluated again by multivariate stepwise linear regression analysis using the parameters described above, peritoneal C_Cr_ (β = 0.278; 95% CI, 0.079–0.476; p = 0.007) and fasting glucose (β = -2.901; 95% CI, -5.420 to -0.381; p = 0.025) were independent variables related to serum irisin levels in PD patients (Table 3). These results suggested that irisin is associated with peritoneal dialysis adequacy and implied that adequate dialysis may improve irisin secretion. Moreover, fasting glucose was inversely associated with serum irisin levels among PD patients.

**Table 3:** Univariate and multivariate regression analysis (outcome: serum irisin levels).

Variables	Unadjusted^a^		Model 1^b^		Model 2^c^		
	Β(95%CI)	P-value	Β(95%CI)	P-value	Β(95%CI)	P-value	
Peritoneal Kt/V_urea_ (per week)	**4.933(0.536 to 9.331)**	**0.029**	**4.933(0.536 to 9.331)**	**0.029**	-		-
Kidney Kt/V_urea_ (per week)	-0.268(-3.229 to 2.694)	0.857	Not selected	-	-		-
Peritoneal C_Cr_ (L/(w*1.73m^2^BSA))	**0.259(0.053 to 0.465)**	**0.015**	-	-	**0.278(0.079 to 0.476)**		**0.007**
Kidney C_Cr_ (L/(w*1.73m^2^BSA))	0.002(-0.049 to 0.053)	0.926	-	-	Not selected		-
Gender(Ref: female)	-1.873(-4.742 to 0.997)	0.196	Not selected	-	Not selected		-
Age (years)	-0.074(-0.199 to 0.052)	0.244	Not selected	-	Not selected		-
Smoker(Ref: non-smoker)	1.610(-3.345 to 6.565)	0.517	-	-	-		-
Drinker(Ref: non-drinker)	1.158(-9.534 to 11.850)	0.829	-	-	-		-
Body mass index (kg/m^2^)	-0.419(-0.931 to 0.093)	0.107	Not selected	-	Not selected		-
Systolic BP (mmHg)	-0.023(-0.076 to 0.030)	0.388	-	-	-		-
Diastolic BP (mmHg)	-0.028(-0.131 to 0.075)	0.589	-	-	-		-
Pulse pressure (mmHg)	-0.043(-0.130 to 0.045)	0.330	-	-	-		-
Fasting glucose (mmol/L)	-2.614(-5.285 to 0.057)	0.055	Not selected	-	**-2.901(-5.420 to -0.381)**		**0.025**
Total cholesterol (mmol/L)	0.804(-0.663 to 2.270)	0.277	-	-	-		-
Triglyceride (mmol/L)	0.700(-0.875 to 2.275)	0.377	-	-	-		-
HDL-C (mmol/L)	1.621(-2.365 to 5.606)	0.418	-	-	-		-
LDL-C (mmol/L)	-0.034(-1.947 to 1.878)	0.971	-	-	-		-
Uric acid (umol/L)	-0.002(-0.020 to 0.016)	0.816	-	-	-		-
ACE inhibitors/ARBs	0.918(-2.177 to 4.012)	0.554	-	-	-		-
Statins	0.340(-3.528 to 4.209)	0.861	-	-	-		-
GNRI	0.050(-0.138 to 0.238)	0.596	-	-	-		-

CI = confidence interval. Ref = reference. Other abbreviations were indicated in Table 1.

^a^ Unadjusted univariate linear regression analysis were performed.

^b^ Variables were tested by multiple stepwise linear regression analysis to determine predictors of serum irisin levels of PD patients. In model 1, peritoneal, kidney Kt/V_urea_, age, gender, BMI and fasting glucose were tested.

^c^ Variables were tested by multiple stepwise linear regression analysis to determine predictors of serum irisin levels of PD patients. In model 2, peritoneal, kidney C_Cr_, age, gender, BMI and fasting glucose were tested.

The final selected variables were presented in the table. Significant results (P<0.05) in **bold.**

### Relationship between irisin levels and bone turnover markers

Intact parathyroid hormone (iPTH) and total alkaline phosphatase (tALP) were bone turnover markers evaluated for CKD–MBD and neither correlated with serum irisin levels. Similarly, no correlations were observed between serum irisin levels and serum levels of corrected calcium, phosphate, or calcium-phosphorus products (Table 4). These results applied to male and female patients individually, as well as the overall PD group. Thus, the association between irisin levels and bone metabolism was not present in PD patients.

**Table 4:** Associations of serum irisin levels and bone turnover markers.

Factors	male(n = 34)		female(n = 25)		total(n = 59)	
	Correlation with irisin (r)	P-value	Correlation with irisin (r)	P-value	Correlation with irisin (r)	P-value
iPTH (pg/ml)	0.154	0.383	-0.148	0.479	0.015	0.909
tALP (U/L)	0.159	0.370	0.192	0.359	0.217	0.099
Corrected calcium (mmol/L)	-0.132 ^a^	0.457	-0.162	0.438	-0.153	0.247
Phosphate (mmol/L)	-0.008	0.965	-0.348	0.088	-0.201	0.127
Calcium-phosphorus product ((mg/ml)^2^)	-0.062	0.728	-0.358	0.078	-0.189	0.151

iPTH = intact parathyroid hormone; tALP = total Alkaline Phosphatase

^a^ Pearson’s correlation analysis was performed. Others were expressed as Spearman rank correlations coefficients.

## Discussion

In this cross-sectional study, we revealed significantly lower serum irisin levels in nondiabetic PD patients compared with age- and sex-matched healthy controls. Multivariate regression analyses also revealed that peritoneal Kt/V_urea_ and peritoneal C_Cr_ were positively correlated with serum irisin levels, suggesting that adequate dialysis may improve irisin secretion. Moreover, fasting glucose levels were inversely correlated with serum irisin levels in PD patients; however, no association between serum irisin levels and HOMA-IR was observed. No association was observed between serum irisin levels and GNRI in PD patients.

Irisin was first identified in 2012 and thought to be involved in energy regulation [1]. Since energy imbalance is prevalent in ESRD, evaluation of circulating irisin levels in ESRD patients helped elucidate the mechanisms of energy dysfunction in ESRD. In the present study, serum irisin levels were significantly lower in nondiabetic PD patients compared with age- and sex-matched healthy controls. These results are in agreement with previous studies showing lower irisin levels in CKD patients, including hemodialysis patients [4–7]. Recently, Lee *et al*. and Rodriguez *et al*. also illustrated that patients undergoing peritoneal dialysis tend to have lower plasma irisin levels compared with healthy controls [8, 9]. While diabetic patients were included in the PD groups in their studies, none was included in the control group. Clinical studies have shown diabetic patients have lower irisin levels than those of subjects with normal glucose tolerance or nondiabetic controls [10, 11]. Kaluzna *et al*. recently also reported irisin levels were lower in ESRD patients compared to controls. However the study had not analyzed the correlation between irisin levels and dialysis adequacy [22]. Since their study included only seven PD patients, it is hard to make the conclusion in PD patients. To address these questions, our study especially included nondiabetic PD patients and controls; therefore, as the first study to identify a correlation between serum irisin and adequacy of peritoneal dialysis. Since irisin is a myokine excreted by muscle, sarcopenia, which is common among ESRD patients, may partially account for this finding. Mid-arm muscle circumference was thought to be an independent predictor of serum irisin levels in PD patients [8]; however, studies have shown that no correlation between irisin levels and anthropometric parameters among the CKD population exist [5, 7]. Discrepancies among study populations, the methods used for assessment of lean body mass and the assay kits used to detect irisin may have contributed to the conflicting conclusions. It is worth noting that indoxyl sulfate, a uremic toxin, can affect the production of FNDC5 and irisin in human skeletal muscle cells [4]. Recently, irisin was found to be secreted by adipose tissue as well, and therefore irisin is an adipo-myokine [23]. To determine whether the expression and secretion of FNDC5/irisin are down-regulated in CKD patients, both subcutaneous adipose tissue and skeletal muscle samples should be assessed. Moreover, the activities of BAT should also be determined in CKD patients to assess whether they are in line with circulating irisin levels.

In this study, peritoneal Kt/V_urea_ or peritoneal C_Cr_ were identified as parameters independently associated with elevated serum irisin levels in PD patients, which suggest that irisin is associated with dialysis adequacy. Lee *et al*. suggested that a higher peritoneal Kt/V_urea_ may result in lower irisin levels, which is different from the results of this study. However, the association between irisin and RRF in that study was determined without adjusting for peritoneal dialysis doses [8]. It should be noted that both RRF and peritoneal dialysis may contribute to the metabolism of irisin. In this study, we addressed the question by performing multivariate regression analyses and found that the more uremic toxins removed by peritoneal dialysis, the higher the circulating irisin levels will be. Our results also suggest that uremic toxins decrease the secretion of irisin in skeletal muscle in a dose-dependent manner [4], and the clearance of uremic toxins may enhance the production of FNDC5/irisin.

Conversely, it has been reported that irisin levels are correlated with insulin resistance among type 2 diabetes mellitus patients [24, 25], and our results also revealed that irisin levels were inversely correlated with fasting blood glucose levels. As insulin resistance is frequently seen in CKD patients, peritoneal dialysis may partially correct this phenomenon [26]. Thus, we suggest that adequate dialysis may improve insulin resistance and increase the levels of circulating irisin. Unfortunately, the circulating irisin levels in PD patients did not correlate with HOMA-IR in our study. Since diabetes patients were excluded from this study, the severity of insulin resistance in our PD patients was minimal.

No association between serum irisin levels and GNRI was observed in this study. As an objective nutritional evaluation score, GNRI predicts the risks of nutrition-related diseases [27]. Our results suggest that serum irisin levels may not indicate PEW in PD patients. To our knowledge, this was the first study to investigate the association between irisin and GNRI. A previous study showed that lower serum irisin levels were associated with sarcopenia in PD patients [8]; however, the different study populations may account for this discrepancy. Importantly, the results of this study should be interpreted with caution, since GNRI is merely a risk index. Machined-based assessments, including dual energy X-ray absorptiometry and body impedance analysis, are validated reference methods for nutritional evaluations and should be used in future studies [28].

There is controversy concerning the association between circulating irisin and fasting glucose levels. While Timmons *et al*. revealed no association in a diabetic population [29], Huh *et al*. found a positive association among healthy women [30]. Our study showed circulating irisin levels were inversely associated with fasting glucose levels in PD patients, and the differences may be explained by the different study populations evaluated. In a study by Kurdiova, glucose decreased the *in vitro* expression of Fndc5 in myotubes in which the levels of Fndc5 mRNA were lower in adipose tissue and plasma in type 2 diabetes patients versus pre-diabetic individuals [31]. As irisin is the cleavage product of FNDC5, the levels of irisin may be down-regulated when Fndc5 expression is reduced.

Increasing evidence suggests that irisin is a vital link between skeletal muscle and bone formation. Previous results were based mainly on postmenopausal osteoporosis [32]. As renal osteodystrophy is characterized by bone remodeling disorders, we explored the association between the expression of bone turnover markers and serum irisin levels in PD patients. No associations were observed, and it should be noted that CKD-MBD is more complicated than osteoporosis. Both tALP and iPTH are bone turnover markers and not completely agreeable with histomorphometric analyses. Thus, bone biopsies are ideal; however, they are invasive and not practical.

The strength of this study was that diabetic patients were excluded from both groups. This is also the first study to explore the relationship between peritoneal dialysis adequacy and irisin after adjusting for RRF. However, this study also has several limitations. This was a cross-sectional, observational study and thus could not provide causal relationships for the findings. The study had a small sample size, and there were confounding factors that were not addressed. Moreover, anthropometry parameters and bone histomorphometric analyses from biopsies were unavailable, and therefore, those correlative analyses were not performed.

Furthermore, the ELISA kits used in our study have been found to reveal lower irisin levels as compared with some other kits in investigations by Choi HY *et al*. and Ebert T *et al* [33, 34]. Inconsistency of circulation irisin levels has cast doubt on the diverse ELISA kits for irisin detection [35]. Albrecht *et al*. have reported the antibodies used in four different commercial ELISA kits had cross-reactions with non-specific serum proteins [36]. But the ELISA kits used in this study has not been tested in their study. The kits we used (Phoenix Pharmaceuticals, Cat EK-067-29) was validated by the immunohistochemistry [37]. Nevertheless, further validation of these ELISA kits is urgently needed.

In conclusion, this study revealed that circulating irisin levels were lower in nondiabetic PD patients compared with healthy controls, and that peritoneal Kt/V_urea_ and creatinine clearance were positively correlated with serum irisin levels in nondiabetic PD patients. These findings are contrary to previous viewpoints that irisin is partially dialyzable. No associations between irisin and HOMA-IR and GNRI were observed, and no association between bone turnover markers and irisin was found among PD patients.

## Supporting information

S1 Checklist STROBE Statement—checklist of items that should be included in reports of observational studies. (DOC)
